# The role of *NPM1* alternative splicing in patients with chronic lymphocytic leukemia

**DOI:** 10.1371/journal.pone.0276674

**Published:** 2022-10-25

**Authors:** Monika Szelest, Marta Masternak, Małgorzata Zając, Michał Chojnacki, Katarzyna Skórka, Joanna Zaleska, Agnieszka Karczmarczyk, Grażyna Stasiak, Ewa Wawrzyniak, Aleksandra Kotkowska, Monika Siemieniuk-Ryś, Joanna Purkot, Edyta Subocz, Edyta Cichocka, Waldemar Tomczak, Daria Zawirska, Krzysztof Giannopoulos

**Affiliations:** 1 Department of Experimental Hematooncology, Medical University of Lublin, Lublin, Poland; 2 Department of Hematology, St. John’s Cancer Centre, Lublin, Poland; 3 Department of Hematology, Medical University of Lodz, Lodz, Poland; 4 Department of Hematology, Military Institute of Medicine, Warsaw, Poland; 5 Independent Public Health Care Center of the Ministry of Internal Affairs and Administration with the Warmian-Masurian Oncology Centre in Olsztyn, Olsztyn, Poland; 6 Department of Hematology, Copernicus Hospital, Torun, Poland; 7 Department of Hematooncology and Bone Marrow Transplantation Unit, Medical University of Lublin, Lublin, Poland; 8 Department of Hematology, Jagiellonian University, Krakow, Poland; Centro de Investigación y de Estudios Avanzados del I.P.N., MEXICO

## Abstract

**Objectives:**

Chronic lymphocytic leukemia (CLL) is a lymphoproliferative disease with heterogeneous clinical course. Recent studies revealed a link between *NOTCH1* mutation and the overexpression of *MYC* and *MYC*-related genes involved in ribosome biogenesis and protein biosynthesis, such as nucleophosmin-1 (*NPM1)*, in CLL cells. In the present study, we aim to evaluate the impact of the *NOTCH1* mutation on the *MYC* and *MYC* induced *NPM1* expression in CLL cells via quantification of their transcripts.

**Methods:**

Using qRT-PCR, we analyzed the levels of *MYC* and three main *NPM1* splice variants in 214 samples collected from CLL patients. We assessed the impact of each splice variant on CLL prognostic markers, including the *IGHV*, *TP53*, *NOTCH1*, *SF3B1*, and *MYD88* mutational status, cytogenetic aberrations, and laboratory features.

**Results:**

Significantly higher levels of *NPM1*.*R1* transcripts in patients with unmutated compared to mutated *IGHV* status were found. The median time to first treatment (TTFT) in patients with a high level of *NPM1*.*R1* was significantly shorter compared to the group with low *NPM1*.*R1* levels (1.5 vs 33 months, p = 0.0002). Moreover, in Multivariate Cox Proportional Hazard Regression Model *NPM1*.*R1* splice variant provided an independent prognostic value for TTFT.

**Conclusion:**

In conclusion, our study indicates the prognostic significance of the level of *NPM1*.*R1* expression and suggests the importance of splicing alterations in the pathogenesis of CLL.

## Introduction

Chronic lymphocytic leukemia (CLL) is a lymphoproliferative disease characterized by the accumulation of morphologically mature and functionally impaired B lymphocytes in the peripheral blood, bone marrow, lymph nodes, liver, and spleen [1]. The heterogeneous course of CLL is reflected in varied survival times, response to treatment, and the dynamics of disease progression [2, 3]. Earlier, prognostication in CLL has been expanded with the addition of markers associated with risk of progression and survival, such as mutational status of the immunoglobulin heavy chain variable region (*IGHV*), chromosomal aberrations, expression of ZAP-70 and CD38, and serum concentrations of β2-microglobulin and lactate dehydrogenase (LDH) [3]. Recently, novel molecular mutations of *TP53*, the neurogenic locus notch homolog protein 1 gene (*NOTCH1*), the myeloid differentiation primary response gene 88 (*MYD88*), and the splicing factor 3B subunit 1 mutation (*SF3B1*) are defined as associated with poor prognosis [4, 5].

Although the identification of these markers provided additional prognostic information, CLL remains an incurable disease, and its varied clinical course requires further research for new prognostic factors, especially those actionable or/and linked to pathomechanism. For instance, Pozzo et al. reported a link between *NOTCH1* mutation and the overexpression of *MYC* and *MYC*-related genes involved in ribosome biogenesis and protein biosynthesis, such as nucleophosmin-1 (*NPM1*) [6]. NOTCH1 is a transmembrane receptor that transduces extracellular signals and acts as a ligand-activated transcription factor. After the interaction of NOTCH1 with DELTA-like or JAGGED ligands expressed by adjacent cells, the extracellular domains of the receptor are cleavaged, which is followed by the release and nuclear translocation of the cytoplasmic intracellular portion of NOTCH1 (NICD). In the nucleus, NICD activates the expression of target genes, such as *MYC*, along with the RBPJ DNA-binding protein [7].

The presence of *NOTCH1* mutations (*NOTCH1*^MUT^) in CLL results in sustained activation of the NOTCH1 signaling pathway and has an impact on prognosis and treatment response in *NOTCH1*^MUT^ CLL cases [7, 8]. The biological mechanisms underlying the proliferative advantages to *NOTCH1*^MUT^ CLL cells remain unexplored and need additional investigation. Nevertheless, Pozzo et al. indicate that *NOTCH1* signaling modulates *NPM1* expression by altered *MYC* transcription [6]. Thus, the overexpression of *NPM1* might contribute to cell proliferation and growth in CLL cases with *NOTCH1* mutation [6].

The *NPM1* gene contains 12 exons and is located on chromosome 5q35. *NPM1* encodes at least seven alternatively spliced isoforms. However, three main splice variants are *NPM1*.*R1*, *NPM1*.*R2*, and *NPM1*.*R3*. Interestingly, none of the protein-coding transcripts contain all 12 exons of the *NPM1* sequence. The predominant isoform *NPM*.*R1* contains 11 exons and lacks exon 10. Another isoform, *NPM1*.*R2*, lacks exons 8 and 10. The *NPM1*.*R3* transcript is translated from exon 1 to 10, thus lacking the domain with a nucleolar localization signal [9].

To date, the critical role of *NPM1* mutations in the pathogenesis and clinical course of acute myeloid leukemia (AML) has been described [10, 11]. However, the impact of *NPM1* alternative splicing on the mechanism driving leukemogenesis has not been determined.

In the present study, we aim to evaluate the impact of the *NOTCH1* mutation on the *MYC* and *MYC* induced *NPM1* expression in CLL cells via quantification of their transcripts. Moreover, due to the different roles of individual splice variants in the functioning of the cell, our study aimed to assess the expression and prognostic significance of selected *NPM1* splice variants, including *NPM1*.*R1*, *NPM1*.*R2*, *NPM1*.*R3*, in patients with CLL.

## Material and methods

### Patient samples

The study included 214 newly diagnosed and untreated patients with CLL. Ethical approval was granted by the Bioethics Committee of the Medical University of Lublin (KE-0254/231/2015). Written informed consent was provided by all participants. The study was performed in line with the principles of the Declaration of Helsinki. The detailed clinical characteristics of patients are presented in Table 1 and S1 Table.

**Table 1:** Clinical characteristics of CLL patients.

**Number of patients**	214	
**Sex**	Female	83
	Male	131
**Median age (years), range**	65, 38–90	
**Rai stage**	0—II	168
	III—IV	28
	Not available	18
***IGHV* mutational status**	Mutated	93
	Unmutated	98
	Not available	23
***NOTCH1* mutational status**	Mutated	35
	Unmutated	171
	Not available	8
***TP53* mutational status**	Mutated	7
	Unmutated	113
	Not available	94
***MYD88* mutational status**	Mutated	6
	Unmutated	188
	Not available	20
***SF3B1* mutational status**	Mutated	5
	Unmutated	54
	Not available	155
**Number of chromosomal alterations (del17, del11q, del13q, del6q, tri12)**	0	47
	1	63
	≥2	13
	Not available	91
**Median β2-microglobulin (μg/L), range**	2.535, 1.33–13.9	
**Median LDH (U/L), range**	384, 163–1497	
**Median WBC (G/L), range**	29.48, 6.16–742	
**Median RBC (T/L), range**	4.46, 2.36–63.96	
**Median HGB (g/dL), range**	13.7, 7.1–16.7	
**Median HCT (%), range**	40.9, 21.2–53.3	
**Median PLT (G/L), range**	178.5, 19.0–2112	
**Median Neut (G/L), range**	4.43, 1.41–27.79	

### Isolation of mononuclear cells, DNA and RNA

Peripheral blood mononuclear cells (PBMC) from 214 blood samples were isolated using Ficoll density gradient centrifugation (Biochrom, Berlin, Germany). For isolation of DNA and RNA, the QIAmp DNA Blood Mini Kit (Qiagen, Hilden, Germany) and the QIAamp RNA Blood Mini Kit (Qiagen, Hilden, Germany) were used, respectively, according to the manufacturer’s protocols. The quality and quantity of the obtained nucleoid acids were quantified with the use of the BioSpec-nano spectrophotometer (Shimadzu, Kyoto, Japan).

The QuantiTect Reverse Transcription Kit (Qiagen, Venlo, Netherlands) was used to perform the reverse transcription reaction, following the manufacturer’s instructions. One microgram of RNA was reverse transcribed into 40 μg of cDNA and diluted (1:1) in water. Five μl of the cDNA sample was used for each qPCR analysis.

The methodology used to determine the mutation status of selected genes (*IGHV*, *TP53*, *SF3B1*, and *MYD88*) has been described in detail earlier [12].

### Droplet digital PCR

For the assessment of the *NOTCH1* mutation status, the QX200 micro drop reader (Bio-Rad, California, USA) and PrimePCR ddPCR Mutation Assays for distinct molecular probes (wild-type and mutation) were used. PCR reaction was performed using 200 ng of template DNA in a final volume of 20 μL. The cycling conditions were: 95°C for 10 min., followed by 40 cycles of 95°C for 30 seconds and 55°C for 60 seconds. Then, the plate was placed into the QX200 micro drop reader (Bio-Rad, California, USA), and obtained data were analyzed with the use of QuantaSoft v1.7.4 Software.

### Primer design and qRT-PCR

To evaluate the *MYC* and *NPM1* splice variants expression at the mRNA level, qPCR was performed using the Fast Start Universal SYBR Green Master kit (Roche Diagnostics, Germany), according to manufacturer’s instructions, with the 7900HT Fast Real-Time PCR System (Applied Biosystems, Life Technologies Corporation, Carlsbad, CA, USA). The sequences of the primers were designed using the Primer 3 software (version 4.0.0) (S2 Table).

The *GAPDH*, coding for glyceraldehyde-3-phosphate dehydrogenase, was used as a reference gene. The TaqMan Universal PCR Master Mix kit (Applied Biosystems, USA) and the TaqMan GAPDH Control Reagents Human Assays (Applied Biosystems, USA) kit were used to evaluate *GAPDH* expression, according to the manufacturer’s guidelines.

The standard curve method was used for the quantity mean values of the transcript’s expression. Standard curves were made based on a conventional PCR reaction with primers specific for *NPM1*.*R1*, *NPM1*.*R2*, and *NPM1*.*R3*, respectively. PCR products were identified using ethidium bromide (Sigma Aldrich, Germany) after gel electrophoresis. The appropriate DNA bands were cut out of the agarose gel and purified using the MiniElute Gel Extraction Kit (Qiagen, Venlo, Netherlands). Concentrations of obtained products were measured using The BioSpec-nano spectrophotometer (Shimadzu, Kyoto, Japan). ENDEMO software was used for the conversion of the splice variant’s concentration to the number of transcript copies. Data were analyzed using SDS 2.3 software (Applied Biosystems, Life Technologies Corporation, Carlsbad, CA, USA).

### Statistical analysis

Statistical analyzes were performed using GraphPad Prism 5.0 (La Jolla, California, USA). Mann-Whitney and Kruskal-Wallis tests were used to assess the differences between the individual subgroups. The correlation of variables was analyzed using Spearman’s rank correlation coefficient. Survival curves were calculated for time to first treatment (TTFT), according to Kaplan-Meier, and compared using the log-rank test. Multivariate Cox Proportional Hazard Regression Model was performed to establish independent prognostic markers for TTFT with the use of R. P-values <0.05 were considered to indicate statistical significance.

As *NPM1* splice variants expression levels are skewed, all analyses were re-performed using normalized data. The analysis of the normalized data is compatible with the analysis of raw data, which was confirmed by proper tests.

## Results

### Expression levels of *NPM1* splice variants in CLL patients

**Fig 1:**
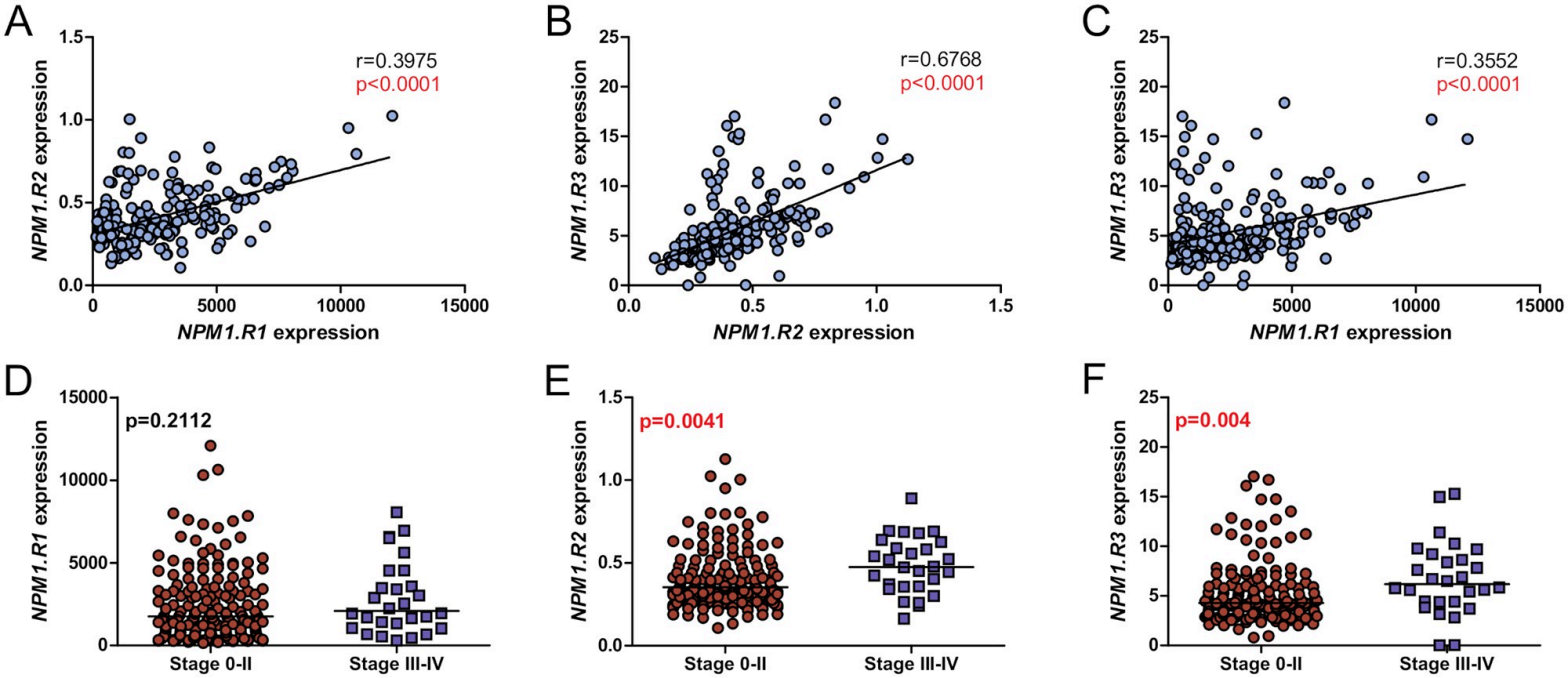
Expression levels of *NPM1* splice variants in CLL patients. (A-C) Correlation between *NPM1*.*R1*, *NPM1*.*R2*, and *NPM1*.*R3* splice variants levels in CLL samples. Correlation plots include Spearman correlation coefficients. (D-F) The comparison of three *NPM1* transcripts levels in CLL samples, according to Rai’s stage. Mann-Whitney P values are included. Each dot represents one sample. Statistically significant p values are indicated in red.

The expression of *NPM1*.*R1*, *NPM1*.*R2*, and *NPM1*.*R3* splice variants was observed in all analyzed samples. The median value of the transcript copy number was 1934.76 for *NPM1*.*R1*, 0.37 for *NPM1*.*R2*, and 4.54 for *NPM1*.*R3*. The median level of expression of each splice variant (*NPM1*.*R1*, *NPM1*.*R2*, *NPM1*.*R3*) defined as an inverse ratio of the difference in cycle threshold (1/ΔCt) was 0.96, 0.74, 0.81 respectively. The levels of analyzed transcripts were correlated (p<0.0001), which was confirmed by respective Spearman correlation coefficient (Fig 1a–1c).

Furthermore, a significantly higher expression of *NPM1*.*R2* and *NPM1*.*R3* splice variants was found in a group of patients in the III and IV stages of the Rai staging system, compared to groups in stages 0-II (*NPM1*.*R2*: median 0.4754 vs 0.3533, p = 0.0041; *NPM1*.*R3*: median 6.168 vs 4.287, p = 0.004;) (Fig 1d–1f).

### The expression of *MYC* and its correlation with *NPM1* splicing variants

**Fig 2:**
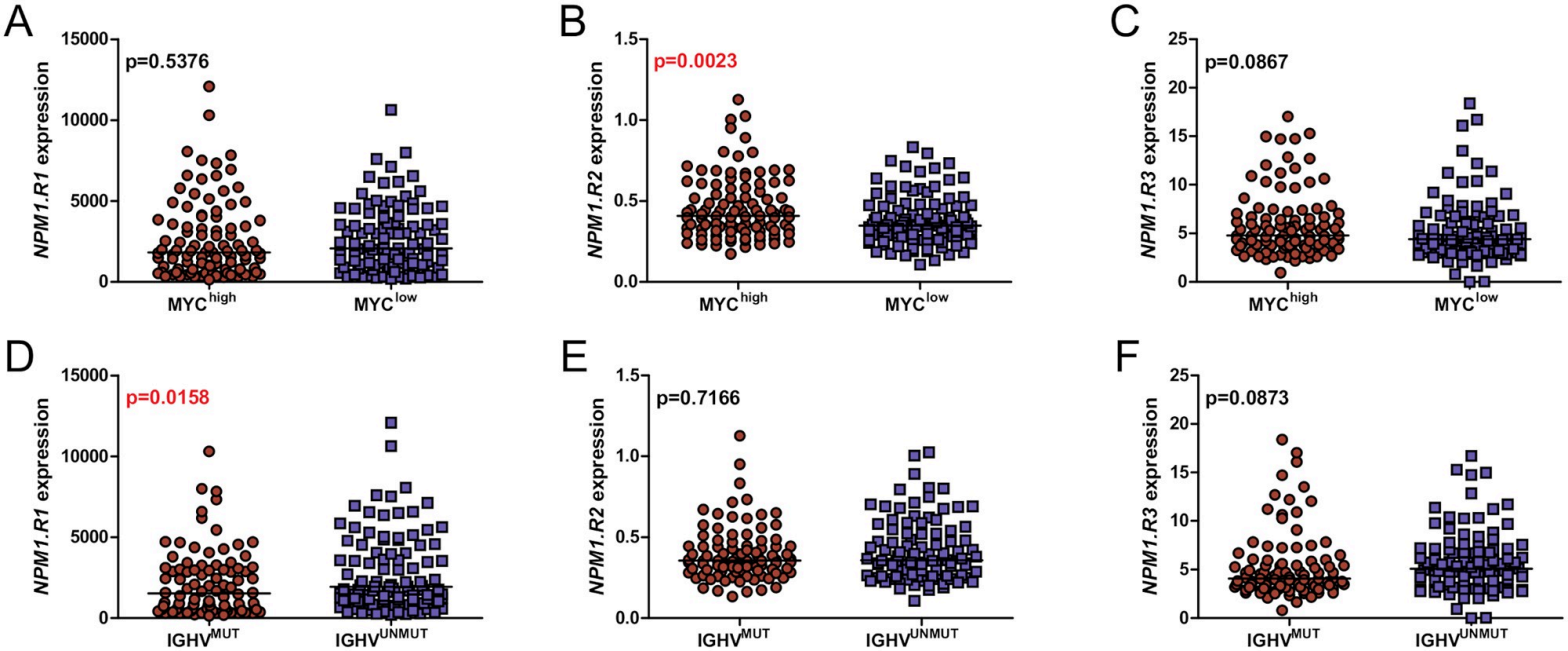
The expression of *NPM1* splicing variants and their correlation with *MYC* level and the *IGHV* mutation status. (A-C) The comparison of three *NPM1* transcripts levels in CLL samples, according to the *MYC* expression level (D-F) and the *IGHV* mutation status. Each dot represents one sample. Mann-Whitney p values are included. Red color indicates statistically significant p values.

The expression of *MYC* was observed in all analyzed samples. The median value of the transcript copy number was 0.08. We found a positive correlation between the levels of *MYC* and *NPM1*.*R2* expression (r = 0.1567, p = 0.0259). Moreover, by dividing patients into two groups with high and low *c-MYC* expression (the cut-off point was set at the median level), we observed a significantly higher expression of *NPM1*.*R2* in the group of patients with a high *MYC* expression in comparison to the group of patients with the low expression level of this isoform (median 0.4077 vs 0.3477, p = 0.0023) (Fig 2a–2c).

As *NOTCH1* mutation might influence the expression of the *MYC*, we aimed to determine the *MYC* expression level regarding *NOTCH1* mutational status. However, there was no significant difference in *MYC* expression in *NOTCHMUT* and *NOTCH1WT* patients.

### The expression of *NPM1* splice variants according to the presence of *IGHV*, *TP53*, *NOTCH1*, *SF3B1*, and *MYD88* mutations

We found a higher expression of the *NPM1*.*R1* splice variant in the unmutated *IGHV* gene (*IGHV*^UNMUT^) group compared to the *IGHV* mutated (*IGHV*^MUT^) group (median for *NPM1*.*R1*: 1934 vs. 1528, p = 0.0158) (Fig 2d–2f). We were not able to observe the difference in the expression of *NPM1*.*R1*, *NPM1*.*R2*, and *NPM1*.*R3* in the groups of *TP53*^MUT^, *NOTCH1*^MUT^, *SF3B1*^MUT^, and *MYD88*^MUT^ patients compared to the wild-type (*TP53*^WT^, *NOTCH1*^WT^, *SF3B1*^WT^, and *MYD88*^WT^) groups, respectively.

### The expression of *NPM1* splice variants regarding the cytogenetic aberrations

**Fig 3:**
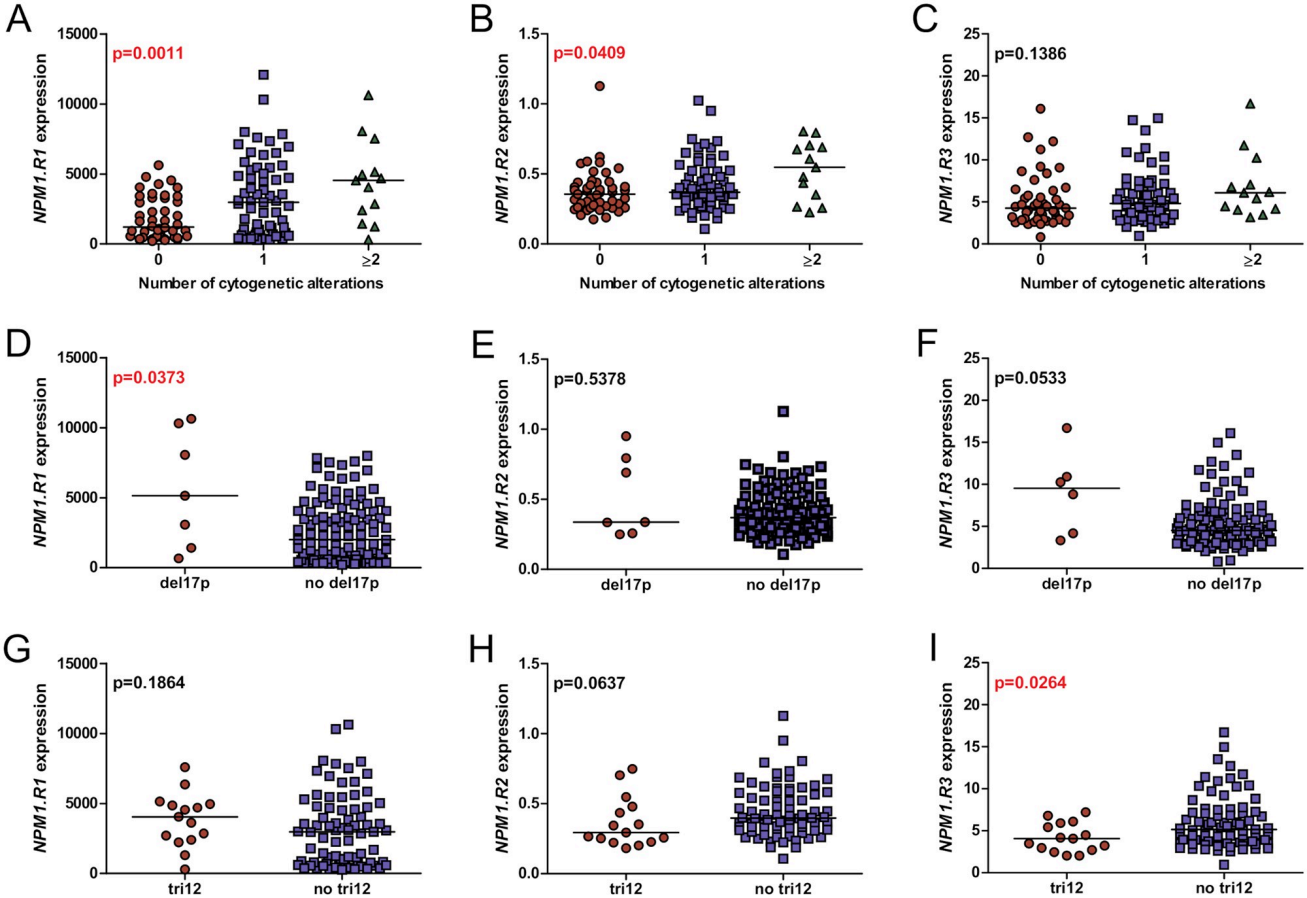
The expression of *NPM1* splice variants regarding the cytogenetic aberrations. The comparison of *NPM1* transcript levels in CLL samples, according to: (A-C) the number of presented cytogenetic alterations, (D-F) the presence of del17, and (G-I) tri12. Mann-Whitney P values are included. Each dot represents one sample. Statistically significant p values are indicated in red.

Analyses of splice variant expression according to the number of cytogenetic abnormalities revealed that patients harboring more changes showed significantly higher copy numbers of *NPM1*.*R1* and *NPM1*.*R2* splice variant compared to patients without cytogenetic aberrations (*NPM1*.*R1*: median 1196 for 0 aberrations, 2965 for 1, 4543 for 2–3; p = 0.0011; *NPM1*.*R2*: median 0.3550 for 0 aberrations, 0.3684 for 1, 0.5472 for 2–3; p = 0.0409) (Fig 3a–3c).

We observed a significantly higher *NPM1*.*R1* expression in patients with del17p compared to the group without del17p (median 5125 vs 2000, p = 0.0373) (Fig 3d–3f). Furthermore, we found a lower expression of *NPM1*.*R3* in patients with trisomy 12 compared to patients with no cytogenetic aberrations (median 4.052 vs 5.156, p = 0.0264) (Fig 3g–3i). We observed no statistically significant differences between the level of *NPM1* splice variants expression and other cytogenetic alterations, such as del11q, del13q, and del6q.

### Impact of *NPM1* splice variants expression on outcome

**Fig 4:**
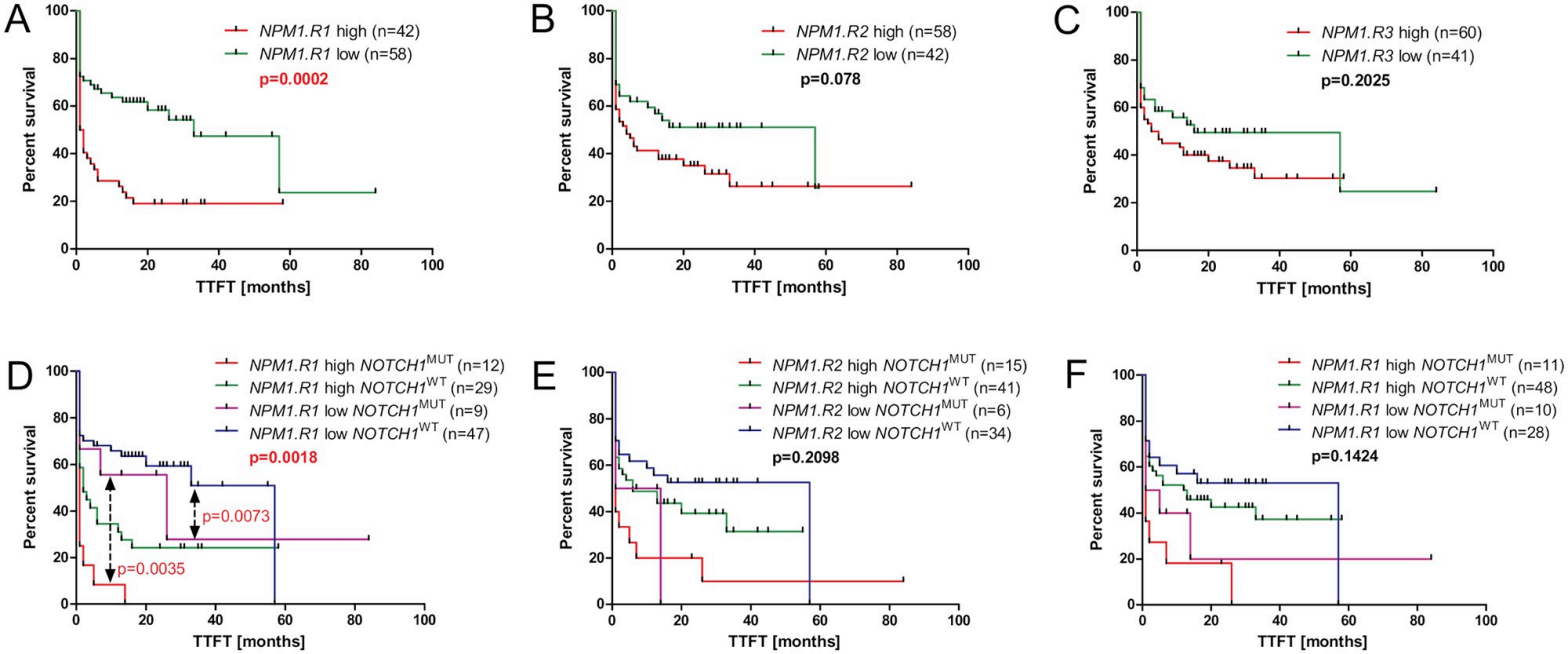
Time to first treatment (TTFT) curves of patients stratified by the combined analysis of *NPM1* variants expression and *NOTCH1* mutation status. Schemes presenting TTFT of CLL patients, according to: (A-C) different *NPM1* transcripts levels and (D-F) both *NPM1* transcripts levels and *NOTCH1* mutation status. Clinically, the prognostic significance in terms of the TTFT was defined as the time from diagnosis to the start of treatment (according to the criteria for treatment proposed by the International Workshop on Chronic Lymphocytic Leukemia). Statistically significant p values are indicated in red.

Correlation of *NPM1* splice variant expression with TTFT was evaluated for groups with high and low expression of *NPM1*.*R1*, *NPM1*.*R2*, and *NPM1*.*R3* (data were dichotomized at the median copy numbers; median 1934.76, 0.37, and 4.54, respectively) (Fig 4a–4c). TTFT was significantly shorter in the group of CLL patients with high *NPM1*.*R1* expression compared to patients with low expression of this splice variant (1.5 vs 33 months, p = 0.0002).

As the analysis of survival curves showed a significantly shorter TTFT in the *NOTCH1*^MUT^ group of patients compared to the group of patients with *NOTCH1*^WT^ (1 vs 16 months, p = 0.0160), we analyzed the clinical relevance of *NPM1*.*R1*, *NPM1*.*R2*, and *NPM1*.*R3* splice variants expression and *NOTCH1* mutation to discriminate the group of CLL patients with the longest TTFT (Fig 4d–4f). In the case of the *NPM1*.*R1* splice variant, *NPM1*.*R1*low/*NOTCH1*^MUT^ group showed significantly longer TTFT compared to *NPM1*.*R1*high/*NOTCH1*^MUT^, and *NPM1*.*R1*high/*NOTCH1*^WT^ group exhibited shorter TTFT in comparison to *NPM1*.*R1*low/*NOTCH1*^WT^ (p = 0.0035 and p = 0.0073, respectively).

Interestingly, in Multivariate Cox Proportional Hazard Regression Model high expression of *NPM1*.*R1* splice variant, unmutated *IGHV*, and stage III-IV in Rai staging system represented an independent factors associated with shorter TTFT (Table 2).

**Table 2:** Multivariate Cox analysis for *NPM1* splice variants and the predictive CLL features (n = 101).

Variable	HR	95% CI	p-value
***NPM1*.*R1* (high vs low expression level)**	1.9532	1.1012–3.464	0.022
***NPM1*.*R2* (high vs low expression level)**	1.2726	0.6458–2.508	0.4862
***NPM1*.*R3* (high vs low expression level)**	0.9962	0.5162–1.922	0.9908
***IGHV* (unmutated vs mutated)**	0.2614	0.1297–0.527	0.0002
***NOTCH1* (mutated vs wild-type)**	1.7946	0.9289–3.467	0.0818
**Stage Rai (stages 0-II vs III-IV)**	2.8095	1.4532–5.432	0.0021

HR—hazard ratio, CI—confidence interval.

### The expression of *NPM1* splice variants among other prognostic groups

We observed a significant positive correlation between *NPM1*.*R1*, *NPM1*.*R2*, *NPM1*.*R3* splice variants expression and β2-microglobulin level (S1 Fig). Furthermore, we found a significant negative correlation between the expression of *NPM1*.*R2* and *NPM1*.*R3* splice variants and LDH concentration (S1 Fig). However, we observed no statistically significant difference in the expression levels of each splice variant in the group ZAP-70(+) compared to ZAP-70(-) and in the group CD38(+) compared to CD38(-).

## Discussion

Recently published data revealed the capability of *NOTCH1* signaling to regulate *NPM1* and the gene encoding ribosomal proteins (RNPs) expression by altering *MYC* transcription [6]. Pozzo et al demonstrated that the gene expression profile in *NOTCH1*^MUT^ CLL cases is highly associated with the *MYC*-induced *NPM1* overexpression [6]. Interestingly, the *NPM1* expression was higher in *NOTCH1*^MUT^ cases irrespective of *IGHV* mutational status [6]. These findings might indicate that the activation of *NOTCH1* signaling, and stimulation of ribosome biogenesis is related to cell proliferation in *NOTCH1*^MUT^ CLL. The proposed mechanism might elucidate the worse clinical outcome of CLL patients with *NOTCH1* mutation. However, *in vitro* studies revealed that NOTCH1 signaling activation promotes NPM1 expression at both transcript and protein levels irrespective of the *NOTCH1* mutation status of CLL cells [9]. Regarding our study, survival curves demonstrated the unfavorable impact of high *NPM1*.*R1* splice isoform expression in both patients with and without *NOTCH1* mutation. However, we observed no differences in *NPM1* splice variants expression levels in groups with distinct *NOTCH1* mutation status. Furthermore, we were not able to detect the difference in *NPM1* transcript levels in *IGHV*MUT and *IGHV*^UNMUT^ CLL regarding the *NOTCH1* mutation status. Therefore, the mechanism of NOTCH1-related overexpression of *NPM1* seems to be largely unexplored.

As mentioned above, the *MYC* expression levels are modulated in the NOTCH1-dependent manner. Thus, the constitutive activation of NOTCH1 signaling, as in CLL with *NOTCH1*^MUT^, might be associated with *MYC*-induced *NPM1* overexpression. Consistent with previous findings, we found no differential transcription of *MYC* between *NOTCH1*^MUT^ and *NOTCH1*^WT^ CLL [6]. However, this might be the result of the fast turnover of *MYC* transcripts [13].

Interestingly, we found a higher *NPM1*.*R2* splice variant expression in the group with high *MYC* expression compared to the group with low *MYC* expression. Moreover, a significant positive correlation between *NPM1*.*R2* and *MYC* expression was observed. However, we found no statistically significant differences between *NPM1*.*R1* and *NPM1*.*R3* expression and *MYC* expression. These data suggest that the impact of *MYC* expression on *NPM1* transcription is more complex and probably is *NOTCH1*-independent.

Alternative splicing promotes protein diversity and plays a key role in the regulation of gene expression. Although abnormal transcripts are usually degraded, the dysfunctional elements of splicing machinery may cause the accumulation of inaccurate splice variants. Thus, the disruption of the mechanism of alternative splicing results in decreased levels of normal proteins or an imbalance in the quantitative ratios among tissue-specific isoforms [14].

Recent studies indicate that alternative splicing may be involved in the pathogenesis and progression of cancer [15]. Hence extensive further research is needed to identify the functional consequences for most of the identified splicing events. Moreover, alternative splicing is the source of novel epitopes, which might be recognized as tumor-specific and therefore become targets for immunotherapy. Using splicing-profile-based personalized combination therapy may improve the outcome for CLL patients. Thus, the identification of individual alternative splicing profiles might also lead to the development of drugs with a greater effectiveness and safety profile.

NPM1 is implicated in both proliferation and growth suppression signal transduction pathways. Moreover, it modulates the activity and stability of key tumor suppressors, such as p53 and ARF. Thus, the double-faced role of NPM1 is reflected in its ability to act as either an oncogene or a tumor suppressor. Notably, NPM1 is involved in key cellular processes, such as ribosome biogenesis, DNA repair, protein chaperoning, and regulation of centrosome duplication, as well as maintenance of genomic stability [16].

To date, no study dedicated to the *NPM1* or its alternative splicing in CLL has been reported. However, the role of alternative splicing of *NPM1* in leukemogenesis was widely studied on AML samples [11, 17]. AML cells have presented the largest number of alternatively spliced events among cancer types [18] The analysis of alternative splicing in bone marrow samples collected from AML patients revealed novel splice variants specific for AML patients in comparison to normal cells, including *NOTCH2* and *FLT3*, thus emerging evidence indicating an important role of splice isoforms in tumor pathogenesis [19].

In our previous study, we highlighted the higher expression of the three main *NPM1* splice variants (*NPM1*.*R1*, *NPM1*.*R2*, and *NPM1*.*R3*) in AML patients with normal karyotype compared to healthy volunteers. Moreover, we reported that the upregulated expression of *NPM1*.*R2* (*NPM1*.*R3* in this study) isoform is associated with longer overall survival compared to patients with *NPM1*.*R2* low expression levels [17]. Furthermore, Handschuh et al. reported increased levels of *NPM1*.*R1*, *NPM1*.*R2*, and *NPM1*.*R3* splice variants in both AML and acute lymphoblastic leukemia (ALL) samples [11]. The analysis of the survival curves of patients with AML indicated that the level of *NPM1* transcript expression has a greater impact on the patient’s prognosis than the *NPM1* mutation status. However, in our study, we found that the expression of *NPM1*.*R1* is associated with poor prognosis, as high expression of this splice variant was observed in the *IGHV*^UNMUT^ group compared to the *IGHV*^MUT^ group of CLL patients. Moreover, a high expression of the *NPM1*.*R1* splice variant was associated with shorter TTFT in CLL patients. Although unmutated *IGHV* status in CLL patients is associated with an adverse outcome, the biological dissimilarity between mutated and unmutated *IGHV* status remains unclear. Nevertheless, higher *NPM1* expression in *IGHV*^UNMUT^ cases in comparison to *IGHV*^MUT^ cases was reported earlier by Rees-Unwin et al. [20] The altered expression of *NPM1* in *IGHV*^UNMUT^ CLL might contribute to changed protein biosynthesis, as *NPM1* regulates the activity of ribosomal proteins.

In accordance with our study, Handschuch et al. observed a high correlation of three main *NPM1* splice variants with each other in both AML and ALL.11 Therefore, this finding indicates a common transcriptional regulation of *NPM1* transcripts. However, the next generation sequencing (NGS)-based transcriptome studies revealed a wide spectrum of the *NPM1* transcripts and the differences in the expression pattern of the *NPM1* splice isoforms might be associated with mutations or aberrant expression of splicing-related genes [11].

Differences in splicing variants’ expression levels may be caused by the presence of mutations in the canonical splice sites or sequences acting as *NPM1* gene transcription regulators. Yoshida et al. proved the presence of mutations in genes regulating alternative splicing in patients with myelodysplastic syndrome [21]. Makishima et al. reported recurrent mutations in genes encoding the spliceosome components (e. g. *SF3B1*, *SFRSF2*, and *UAF35*) [22].

*SF3B1* mutations are associated with splicing alterations, thus affecting various cellular functions, such as DNA damage response, apoptosis, and the *NOTCH1* signaling pathway. Mutated *SF3B1* is associated with poor outcomes in CLL and is found more often in refractory CLL [23]. Mutation in *SF3B1* dysregulates various cellular functions by splicing pattern alterations and changing gene expression [24]. Furthermore, *SF3B1* mutation causes numerous transcript-level aberrations on a wide range of genes at relatively low potency per gene, thus modulating CLL biology [25]. Yin et al. revealed a contributory driving role of mutated *SF3B1* in CLL progression, as the combination of *SF3B1* mutation and *ATM* deletion restricted to B-cells resulted in CLL-like disease in mouse model due to increased genomic instability [25]. Therefore, *SF3B1* mutations are associated with enhanced DNA damage and impaired response to DNA damage. Interestingly, in contrast to the widespread intron retention observed in most tumors, intronic splicing was found to be characteristic for CLL cells compared with normal B-cells [18, 26]. Thus, as a consequence of these events, the expression of certain isoforms and the function of particular proteins may be altered. However, in our study, we found no statistically significant differences in the *NPM1* splice variants levels regarding the *SF3B1* mutational status. Nevertheless, we found only 5 *SF3B1*^MUT^ cases among 59 analyzed samples.

Interestingly, we observed a significantly higher expression of the *NPM1*.*R2* and *NPM1*.*R3* splice variants in a group of patients in the III and IV stages of the Rai staging system compared to the group in the 0-I stages. Emerging evidence indicates an essential role of the *NPM1* overexpression in distinct human neoplasms [27–29]. However, most of previous reports are focused on gene or protein level of NPM1, without distinguishing different protein-coding isoforms. Thus, while the function of the longest and the most frequently transcribed *NPM1*.*R1* splice variant is well studied, the biological significance of other NPM1 transcripts and their contribution to leukemia maintenance and progression remains largely unexplored.

Although the *NPM1*.*R2* transcript comprises 10 exons, with exons 8 and 10 missing, the encoded protein is similar to the predominant *NPM1*.*R1* isoform. Therefore, despite slightly different structures, these two proteins might carry out similar functions in the cell. Of note, high expression level of NPM1 has been described as an early marker of proliferative activity in leukemia-derived cell lines, as it precedes the S-phase of the cell cycle [30]. On the other hand, *NPM1*.*R3* isoform preferentially localizes in the nucleoplasm, due to the lack of the domain required for nucleolar localization. Therefore, this truncated NPM1 splice variant resembles mutated NPM1. The high level of *NPM1*.*R3* indicates its important role in malignant cells. For instance, the biological impact of *NPM1*.*R3* might be reflected in aberrant interaction with nuclear proteins, which in turn could influence signaling pathways and eventually provide proliferative advantages to CLL cells. For instance, it was reported that the abnormal cytoplasmic accumulation of NPM1 is associated with immediate upregulation of homeobox (*HOX)* genes, which promotes leukemic cell proliferation [31]. The cytoplasmic mis-localization of the shortened *NPM1* variant might influence the leukemic burden, thus providing the rationale for application of the nuclear export inhibitors in cancer with either mutated *NPM1* or high expression level of *NPM1*.*R3*.

## Conclusions

In conclusion, our study indicates the prognostic significance of *NPM1*.*R1* splice variant expression in CLL cases. Furthermore, we showed the association between *NPM1*.*R1* overexpression and adverse outcomes in CLL patients. Regarding our studied group, including patients with different cytogenetic aberrations and mutation status of distinct disease-related genes, the *NPM1*.*R1* transcription level affected patient outcome more than *NOTCH1* mutation. The significant relationship between the expression of *NPM1* splice variants and important prognostic factors for CLL indicates that alternative splicing may be a mechanism that plays an important role in the pathogenesis of CLL.

## Supporting information

S1 Table Clinical characteristics of CLL patients regarding the cytogenetic alterations and ZAP-70 and CD38 expression. (DOCX)

S2 Table Primers used in qPCR analysis. (DOCX)

S1 Fig The comparison of the *NPM1* transcripts expression in CLL samples, according to. (A-C) levels of β_2_-microglobulin and (D-F) lactate dehydrogenase (LDH). Each dot represents one sample. Correlation plots include Spearman correlation coefficients. Statistically significant p values are indicated in red.
(TIF)
