# Supplementary material for: The role of NPM1 alternative splicing in patients with chronic lymphocytic leukemia
Source: PLoS One. 2022 Oct 25;17(10):e0276674. doi: 10.1371/journal.pone.0276674 (PMC9595542; doi:10.1371/journal.pone.0276674)
Supplement: S1 Table — (DOCX) [file pone.0276674.s001.docx]

| **Del17p** | Presented | 7 |
| --- | --- | --- |
|  | Analyzed cases | 121 |
| **Del11q** | Presented | 13 |
|  | Analyzed cases | 122 |
| **Del13q** | Presented | 49 |
|  | Analyzed cases | 93 |
| **Tri12** | Presented | 15 |
|  | Analyzed cases | 93 |
| **Del6q** | Presented | 6 |
|  | Analyzed cases | 94 |
| **ZAP-70** | Positive (≥20%) | 63 |
|  | Negative (≤20%) | 108 |
|  | Not available | 43 |
| **CD38** | Positive (≥30%) | 51 |
|  | Negative (≤30%) | 126 |
|  | Not available | 37 |
