# Supplementary material for: The role of NPM1 alternative splicing in patients with chronic lymphocytic leukemia
Source: PLoS One. 2022 Oct 25;17(10):e0276674. doi: 10.1371/journal.pone.0276674 (PMC9595542; doi:10.1371/journal.pone.0276674)
Supplement: S2 Table — (DOCX) [file pone.0276674.s002.docx]

| **Transcript or splice variant** | **Transcript ID** | **Protein ID** | **Primer** | **Sequence** |
| --- | --- | --- | --- | --- |
| ***NPM1.R3***  w/o ex. 11 and 12 | NM_001037738.3 | NP_001032827.1  (259 aa) | F | 5’-AGAAAAAGCGCATTGAacag-3’ |
|  |  |  | R | 5’-atgggtggcaaatcagaaaa-3’ |
| ***NPM1.R1***  w/o ex. 10 | NM_001355006.2 | NP_001341935.1  (294 aa) | F | 5’-CTGACCAAGAGGCTATTCAAGA-3’ |
|  |  |  | R | 5’-gcattataaaaaggacagccaga-3’ |
| ***NPM1.R2***  w/o ex. 8 and 10 | NM_199185.4 | NP_954654.1  (265 aa) | F | 5’-CGCCAGTGAAGAAAGGACAA-3’ |
|  |  |  | R | 5’-CCGGAAGCAATTCTTCACAT-3’ |
| ***MYC*** | NM_002467.6 | NP_002458  (454 aa) | F | 5’-TCGGATTCTCTGCTCTCCTC-3’ |
|  |  |  | R | 5’- GAGCCTGCCTCTTTTCCAC-3’ |
| ***GAPDH*** | NM_002046.7 | NP_002037  (335 aa) | F | 5’- TGCACCACCAACTGCTTAGC-3’ |
|  |  |  | R | 5’- GGCATGGACTGTGGTCATGAG-3’ |
