# Supplementary figures and images for: The role of NPM1 alternative splicing in patients with chronic lymphocytic leukemia
Source: PLoS One. 2022 Oct 25;17(10):e0276674. doi: 10.1371/journal.pone.0276674 (PMC9595542; doi:10.1371/journal.pone.0276674)

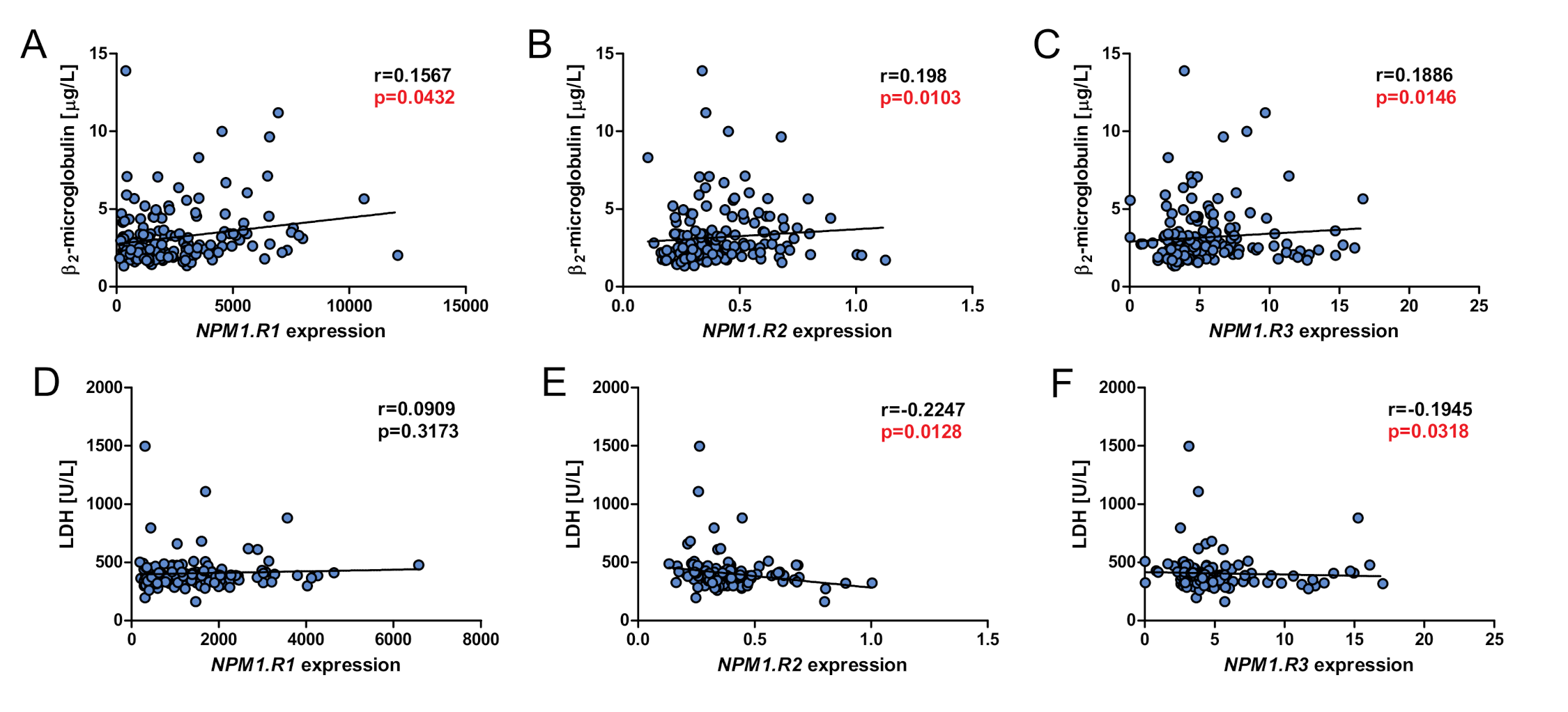

Supplement: S1 Fig — (A-C) levels of β2-microglobulin and (D-F) lactate dehydrogenase (LDH). Each dot represents one sample. Correlation plots include Spearman correlation coefficients. Statistically significant p values are indicated in red. (TIF) [file pone.0276674.s003.tif]
